# Measuring outcomes in allergic rhinitis: psychometric characteristics of a Spanish version of the congestion quantifier seven-item test (CQ7)

**DOI:** 10.1186/1477-7525-9-14

**Published:** 2011-03-10

**Authors:** Antonio Valero, Joaquim Mullol, Michael Herdman, Maria-José Rosales

**Affiliations:** 1 Allergy Unit, Pneumology and Allergy Department, Hospital Clínic, Villarroel 170, Barcelona 08036, Spain; 2 Rhinology Unit & Smell Clinic, ENT Department, Hospital Clínic, Villarroel 170, Barcelona 08036, Spain; 3 CIBERES, Barcelona, Spain; 4 Global Allergy & Asthma European Network; 5 Insight Consulting & Research, Cami Ral 266, Mataró, Spain; 6 CIBER in Epidemiology and Public Health (CIBERESP), Spain; 7 Health Services Research Unit, IMIM-Hospital del Mar, Barcelona, Spain; 8 Medical Affairs Manager, MSD España, Josefa Valcárcel 38, Madrid, Spain

## Abstract

**Background:**

No control tools for nasal congestion (NC) are currently available in Spanish. This study aimed to adapt and validate the Congestion Quantifier Seven Item Test (CQ7) for Spain.

**Methods:**

CQ7 was adapted from English following international guidelines. The instrument was validated in an observational, prospective study in allergic rhinitis patients with NC (N = 166) and a control group without NC (N = 35). Participants completed the CQ7, MOS sleep questionnaire, and a measure of psychological well-being (PGWBI). Clinical data included NC severity rating, acoustic rhinometry, and total symptom score (TSS). Internal consistency was assessed using Cronbach's alpha and test-retest reliability using the intraclass correlation coefficient (ICC). Construct validity was tested by examining correlations with other outcome measures and ability to discriminate between groups classified by NC severity. Sensitivity and specificity were assessed using Area under the Receiver Operating Curve (AUC) and responsiveness over time using effect sizes (ES).

**Results:**

Cronbach's alpha for the CQ7 was 0.92, and the ICC was 0.81, indicating good reliability. CQ7 correlated most strongly with the TSS (r = 0.60, p < 0.01), the PGWBI general health dimension (r = 0.56, p < 0.01), and the MOS Sleep scale 'sleep short of breath' dimension (r = 0.49, p < 0.01). Correlations with acoustic rhinometry were generally low. The instrument discriminated well between NC severity groups (ES 0.33-2.07) and AUC was 0.93, indicating excellent sensitivity and specificity. The measure was responsive to change (ES = 1.1) in patients reporting improvement in NC.

**Conclusions:**

The Spanish version of the CQ7 is appropriate for detecting, measuring, and monitoring NC in allergic rhinitis patients.

## Findings

### Objectives

Nasal congestion (NC) has been described as one of the most troublesome symptoms for patients with allergic rhinitis (AR) and is associated with poorer sleep, mood, and productivity [1,2]. A new tool to measure patient experience of NC is the Congestion Quantifier Seven-Item test (CQ7) which was developed recently in the United States [3]. The CQ7 was originally developed as a screening tool to identify patients with NC potentially requiring treatment and the original version was shown to have excellent reliability, validity, sensitivity and specificity, and responsiveness [3,4]. The objectives of the present study were to assess the reliability, validity, sensitivity and specificity, and responsiveness of a version of the CQ7 for use in Spain.

### Cultural adaptation and validation study

The CQ7 was adapted into Spanish for Spain following a process of cultural adaptation based on international recommendations, which included translation into Spanish by two independent translators, back-translation into English, and cognitive debriefing in 10 patients with AR and NC [5]. The psychometric properties of the Spanish version were then tested in an observational, prospective, multicenter study carried out in the Allergology departments of 17 Spanish hospitals. The majority of patients made one study visit but in some centers they made two (baseline and follow-up at one month) to examine test-retest reliability and responsiveness.

The main study group (N = 166) were outpatients with NC and a clinical diagnosis of intermittent or persistent AR as defined in the ARIA (Allergic Rhinitis in Asthma) guidelines [6]. Patients could be treated or untreated for AR and/or NC at the time of inclusion. Control subjects (N = 35) had to be without NC on inclusion and there was no requirement for a diagnosis of AR.

Variables collected at baseline were: age, gender, educational level, time from diagnosis of allergic rhinitis, frequency and duration of nasal symptoms associated with AR, presence of other diseases, treatment for AR, overall NC severity (clinician and patient ratings), and acoustic rhinometry (in selected centres). In acoustic rhinometry testing (SER 2000, Rhinometrics, Lynge, Denmark), nasal volume (V_0-7_) was assessed from the nostril to 7 cm and minimum cross-sectional area (mCSA) was assessed in both nostrils. Clinicians also completed the Total Symptom Score (TSS) for all patients. The TSS consists of 5 questions measuring AR symptoms and provides an overall score raging from 0 (no symptoms) to 15 (very severe symptoms).

Patients completed the Spanish version of the Congestion Quantifier Seven-Item Test (CQ-7), the Psychological General Well -Being Index (PGWBI) [7], and the Medical Outcomes Study Sleep Scale (MOS Sleep) [8,9]. The CQ-7 consists of 7 items answered on a scale from 0 (never) to 4 (always) with a total score ranging from 0 (no nasal congestion) to 28 (worst nasal congestion). The overall score is a simple summation of the individual item scores. The time frame for all instruments was the previous week and all had been adapted and validated for use in Spain [10,11].

Patients who attended the follow-up visit completed a global rating of change item. The latter was used to measure perceptions of change in NC from baseline on a scale with 13 response options ranging from 'A very great deal better' to 'A very great deal worse'.

Ethics approval for the study was provided by the Ethics Committee of the Hospital Clínic in Barcelona and all patients taking part in the study provided written informed consent to participate.

### Statistical analysis

The *feasibility *of the Spanish version of the CQ7 was assessed by examining the proportion of missing responses and the proportion of patients who found the instrument easy to use. The proportion of patients with the worst and best possible scores was calculated to estimate floor and ceiling effects, while internal consistency (reliability) was assessed using Cronbach's alpha coefficient [12]. Test-retest reliability was assessed by computing the intraclass correlation coefficient (ICC) in patients reporting no or only minimal change on the global rating of change item [13]. Convergent validity [13] was tested by analyzing the extent to which CQ7 scores demonstrated logical relationships with other outcomes measures (PGWBI, MOS Sleep, TSS, acoustic rhinometry) and known groups' validity was tested by determining the ability of the instrument to discriminate between groups defined by different categories of severity on the NC severity rating item (according to both patient and clinician overall ratings). T tests and effect sizes were used to analyze the extent of differences between groups. Sensitivity and specificity were evaluated using receiver operating characteristic (ROC) curve analysis to determine whether the questionnaire discriminated between patients with NC and controls. Responsiveness to change was assessed by determining the extent to which the instrument captured change in health status in patients reporting improvement or worsening on the global rating of change item. Change over time was analyzed using t tests and effect sizes. For all analyses, the level of statistical significance was set at 0.05 and all analyses were performed in version 13.0 of SPSS.

## Results

A total of 201 individuals participated in the validation study (166 patients with NC and 35 controls without NC). Sample characteristics are shown in Table 1. The study population was relatively young with a mean age of 34.3 years, and a slight predominance of women.

**Table 1:** Sample characteristics at baseline: controls, patients with nasal congestion, and overall

	Patients	Control	
	(n = 166)	(n = 35)	P*
Age, mean (SD), years	33.9 (11.9)	36.1 (11.7)	NS
Male, n (%)	78 (47.3%)	8 (22.9%)	0.008
			
Highest educational level, n (%)			
*No formal education*	2 (1.21%)	0 (0.00%)	0.009
*Primary*	25 (15.2%)	5 (14.3%)	
*Secondary*	78 (47.3%)	7 (20.0%)	
*Post-secondary*	60 (36.4%)	23 (65.7%)	
			
Physician rating of NC severity, n (%)			
*None*	2 (1.2%)	33 (94.2%)	<0.001
*Mild*	56 (33.7%)	2 (5.8%)	
*Moderate*	53 (31.9%)	0 (0.0%)	
*Severe*	43 (25.9%)	0 (0.0%)	
*Very severe*	12 (7.2%)	0 (0.0%)	
			
Patient rating of NC severity, n (%)			<0.001
*None*	2 (1.2%)	33 (94.3%)	
*Very Mild*	16 (9.6%)	1 (2.9%)	
*Mild*	40 (24.1%)	1 (2.9%)	
*Moderate*	53 (31.9%)	0 (0.0%)	
*Severe*	43 (25.9%)	0 (0.0%)	
*Very severe*	12 (7.2%)	0 (0.0%)	
			
Treatment			<0.001
*Topical corticosteroids*	72 (43.4%)	2 (5.7%)	
*Oral corticosteroids*	1 (0.8%)	0 (0%)	
*Topical antihistamines*	1 (0.6%)	0 (0%)	
*Oral antihistamines*	57 (34.3%)	2 (5.7%)	
			
Acoustic rhinometry,** mean (SD)			
*Nasal volume (V*_*0-7*_*)*	18.6 (8.4)	-	-
*mCSA*	0.93 (0.45)	-	-
CQ7 score, mean (SD)	15.9 (5.2)	3.7 (4.3)	<0.001
PGWBI score, mean (SD)	88.3 (13.3)	97.1 (14.3)	<0.001
MOS Sleep score, mean (SD)			
*Sleep problems index II*	35.1 (17.5)	22.3 (15.4)	<0.001

NC: nasal congestion; TSS: Total Symptom Score; mCSA: minimum cross-sectional area; CQ7: Congestion Quantifier 7 item; PGWBI: Psychological General Well-Being Index; MOS: Medical Outcomes Study

There were no missing responses on any of the CQ7 items in any of the study visits (see Table 2). The majority of respondents (controls and patients) found the questionnaire 'easy' (33.3%) or 'very easy' (56.2%) to complete. Ceiling and floor effects (1.2% and 0.6%, respectively) were very small in the patient sample. Internal consistency was very satisfactory in the overall sample (Cronbach's alpha of 0.92) and test-retest reliability assessed in patients reporting no or only minimal change in NC at follow-up (n = 24) was also acceptable (ICC of 0.81).

**Table 2:** Score distributions, internal consistency, and missing responses on the CQ7: overall sample and patient and control groups (baseline visit).

	CQ7: overall sample	CQ7: Patients	CQ7: Controls
	(n = 201)	(n = 166)	(n = 35)
Missing responses*, n	0	0	0
Theoretical score range	0 - 28	0 - 28	0 - 28
Observed score range	0 - 25	0 - 25	0 - 16
Mean (SD) score on CQ7	14.5 (5.6)	15.9 (5.2)	3.6 (4.3)
Cronbach's alpha	0.92	0.86	0.89
Ceiling effect (%) ^a^	1.0	1.2	2.9
Floor effect (%) ^b^	7.0	0.6	37.1

*Number and proportion of respondents with at least one missing response on the CQ7

^a^% of respondents with the highest (worst) possible score on the CQ7

^b^% of respondents with the lowest (best) possible score on the CQ7

Correlations between the CQ7 and other outcome measures showed the expected patterns (Table 3). The CQ7 score correlated most highly with the TSS (r = 0.60, p < 0.0001), though moderate to high correlations were also seen with the vitality (*r *= 0.33, p < 0.0001) and general health (r = 0.56, p < 0.0001) dimensions of the PGWBI. Correlations with the MOS Sleep questionnaire were highest for dimensions related with breathing difficulties, i.e. the 'sleep short of breath/headache', 'sleep disturbance' and 'snoring' dimensions (correlations of r = 0.49, 0.47, and 0.35, respectively; p < 0.0001). Correlations with acoustic rhinometry values were generally low, particularly at the first visit.

**Table 3:** Pearson correlation coefficients at baseline between CQ7, PGWBI (overall and dimensions), MOS Sleep scale (overall and by dimension), acoustic rhinometry results, and Total Symptom Score

	Correlation coefficient	P value
**PGWBI**		
Anxiety	-0.27	0.0001
Depression	-0.21	0.002
Positive mood	-0.37	0.0001
Vitality	-0.33	0.0001
Self-control	-0.19	0.006
General health	-0.56	0.0001
Overall score	-0.40	0.0001

**MOS Sleep scale**		
Sleep disturbance	0.47	0.0001
Snoring	0.35	0.0001
Sleep short of breath (headache)	0.49	0.0001
Sleep adequacy	-0.21	0.003
Sleep somnolence	0.23	0.001
Sleep problems index I	0.37	0.0001
Sleep problems index II	0.49	0.0001

**Acoustic rhinometry**		
Nasal volume (V_0-7_)	-0.07	NS
mCSA	-0.21	0.066

**TSS**	0.60	0.0001

PGWBI: Psychological General Well-Being Index; MOS: Medical Outcomes Study; mCSA: minimum cross-sectional area; TSS: Total Symptom Score.

**Figure 1:**
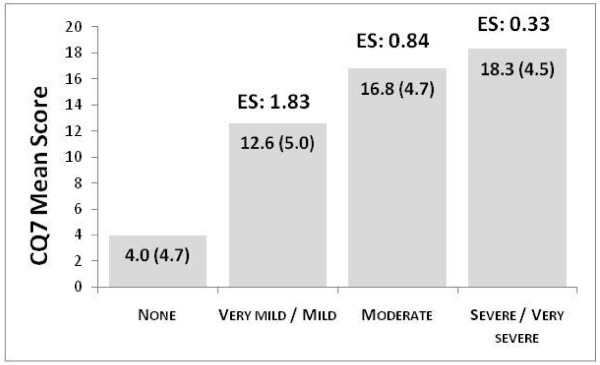
**Bar chart**. ES: Effect size. Differences in mean score between all categories were statistically significant (p < 0.05; Tukey correction for multiple comparisons) except between Moderate and Severe/Very severe.

The CQ7 discriminated well between groups defined by NC severity (Figure 1). Between-group effect sizes using clinician-rated NC severity ranged from 0.33 to 1.83 which would represent small and large effect sizes, respectively. Similar results were observed using patient self-ratings of overall NC severity.

The instrument showed good sensitivity and specificity for detecting cases of nasal congestion with an area under the ROC curve over 0.90 (AUC = 0.948, IC95% [0.912 - 0.985]; p < 0.001). The optimum cut-point for discriminating between cases and non-cases on the CQ7 was 7 points, which gave a sensitivity of 94% and a specificity of 85.7%.

In 39 patients (55.7%) who reported improvement on the global rating of change item the between visit difference in mean CQ7 scores was statistically significant (p < 0.001) with an effect size of 1.1, representing a large effect size (Table 4).

**Table 4:** Change in CQ7 scores after 1 month based on patient global rating of change in nasal congestion

	CQ7					
Change in nasal congestion	Baseline	1 month	Difference	p (*)	Effect Size	p(**)
Improved (n = 39)	15.3 (5.2)	9.5 (6.1)	5.8 (5.8)	0.000	-1.11	<0.001
Stable (n = 24)	12.2 (5.4)	10.6 (6.2)	1.5 (3.4)	0.035	-0.29	
Worse (n = 10)	14.8 (6.3)	18.1 (4.3)	-3.3 (6.2)	0.124	0.52	
Total (n = 73)	14.2 (5.6)	11.1 (6.5)	3.2 (6.0)			

*p value for difference between scores at the two visits

**p value for the difference in change scores between the 3 groups

## Conclusions

The results of the present study show that the Spanish version of the CQ7 has excellent psychometric properties which were similar to or, in some cases, superior to those shown by the original version. The great majority of patients found the instrument easy to complete which, coupled with the very low rate of missing responses, indicates excellent acceptability. Likewise, the instrument discriminated well between patients defined by level of clinical severity and correlated in the way expected with other outcome measures. Sensitivity and specificity were excellent and the instrument appeared to be very responsive to change.

The results observed here showed that the Spanish version of the instrument had psychometric properties which were similar to those of the original version. That version also had high reliability coefficients (Cronbach's alpha of 0.93 and an ICC of 0.85), discriminated well between patients and controls (AUC of 0.97), and correlated well with the MOS Sleep scale (correlations were slightly stronger than those observed here, ranging from 0.21 to 0.67). The authors of that instrument also found that a cut point of 7 points would optimize sensitivity and specificity [3]. The similarity of the results adds to the robustness of our findings as they are indicative of an instrument that works consistently across these two languages/cultures.

Interestingly, correlations between CQ7 scores and acoustic rhinometry at baseline were non-existent or minimal, while considerably stronger correlations were observed at the second study visit, though these were still low to moderate. Nevertheless, we did not expect a very much stronger correlation as the two indicators measure substantially different things; rhinometry is a biological parameter measuring nasal geometry whereas the CQ7 measures the subjective perception of air through the nasal cavities and the impact of NC on activities. The stronger correlation with the mCSA could suggest that the aspects measured by the CQ7 are more closely related with the sensation of nasal obstruction than with nasal volume.

Study limitations include the small number of respondents in the control group and, in particular, the fact that the control group had a higher proportion of males and was better educated. This might have led to better scores on the CQ7 as education and being male are often associated with higher scores on patient reported outcome measures. The difference in score between the two groups may have been smaller with a larger control group with more similar characteristics to the patient group, though the difference would likely remain substantial. Although the method of assessing test-retest reliability employed here is commonly used in assessing PRO instruments, the small number of patients included in this analysis and the fact that only patients reporting no or minimal change were included may have introduced a selection bias. This characteristic should be tested in larger samples in the future.

Taking into account the study limitations, we nevertheless believe that our findings indicate that the Spanish version of the CQ7 questionnaire is a practical, reliable, and valid screening tool to detect and monitor cases of nasal congestion in allergic rhinitis patients.

## Abbreviations

NC: nasal congestion; CQ7: Congestion Quantifier 7 item; MOS: Medical Outcomes Study; PGWBI: Psychological General Well-being Index; TSS: Total Symptom Score; ICC: intraclass correlation coefficient; AUC: Area under the Receiver Operating Curve; ES: effect size; AR: allergic rhinitis; HRQOL: health-related quality of life; ARIA: Allergic Rhinitis and its Impact on Asthma; mCSA: minimum cross-sectional area; ROC: receiver operating characteristic; ANOVA: analysis of variance.

## Competing interests

AV, JM, and MH received funding from Schering-Plough, Spain for their involvement in this study. MJR is an employee of Schering-Plough, Spain. Schering-Plough, Spain financed the present manuscript, including the article-processing charge.

## Authors' contributions

AV, JM, and MH designed the study. AV and JM were the principal study investigators. MH designed the statistical analyses and drafted the manuscript. All authors contributed substantially to the design of the study, the interpretation of the results, and the editing of the manuscript. All authors read and approved the final manuscript.
